# Evidence for Horizontal Transmission of Secondary Endosymbionts in the *Bemisia tabaci* Cryptic Species Complex

**DOI:** 10.1371/journal.pone.0053084

**Published:** 2013-01-07

**Authors:** Muhammad Z. Ahmed, Paul J. De Barro, Shun-Xiang Ren, Jaco M. Greeff, Bao-Li Qiu

**Affiliations:** 1 Department of Entomology, South China Agricultural University, Guangzhou, People's Republic of China; 2 Department of Genetics, University of Pretoria, Pretoria , South Africa; 3 CSIRO Ecosystem Sciences, Brisbane, Queensland, Australia; Wageningen University, The Netherlands

## Abstract

*Bemisia tabaci* (Hemiptera: Aleyrodidae) is a globally distributed pest composed of at least 34 morphologically indistinguishable cryptic species. At least seven species of endosymbiont have been found infecting some or all members of the complex. The origin(s) of the associations between specific endosymbionts and their whitefly hosts is unknown. Infection is normally vertical, but horizontal transmission does occur and is one way for new infections to be introduced into individuals. The relationships between the different members of the cryptic species complex and the endosymbionts have not been well explored. In this study, the phylogenies of different cryptic species of the host with those of their endosymbionts were compared. Of particular interest was whether there was evidence for both coevolution and horizontal transmission. Congruence was observed for the primary endosymbiont, *Portiera aleyrodidarum*, and partial incongruence in the case of two secondary endosymbionts, *Arsenophonus and Cardinium* and incongruence for a third, *Wolbachia*. The patterns observed for the primary endosymbiont supported cospeciation with the host while the patterns for the secondary endosymbionts, and especially *Wolbachia* showed evidence of host shifts and extinctions through horizontal transmission rather than cospeciation. Of particular note is the observation of several very recent host shift events in China between exotic invader and indigenous members of the complex. These shifts were from indigenous members of the complex to the invader as well as from the invader to indigenous relatives.

## Introduction

A large number of herbivorous insects including the phloem feeding insects of the Hemiptera suborder Sternorrhyncha (aphids, whiteflies, psyllids, scales and mealybugs) harbour endosymbiotic bacteria [1]. The phloem is nutrient deficient, and the metabolites produced by some of these bacteria fortify their diet [2]. These bacteria, referred to as the primary endosymbionts (P-endosymbionts), are confined to specialized host cells called bacteriocytes (or mycetocytes) which form the bacteriome [3] and are transmitted vertically from mother to offspring [4]. In addition, insects have more recently acquired a number of endosymbiotic bacteria which in many cases live outside the bacteriocytes [3]–[5]. These bacteria are designated as secondary endosymbionts (S-endosymbionts). Their relationship with the host may be either facultative or obligatory and as well as being transmitted vertically to offspring, may also be transmitted horizontally through either direct and indirect contact with other infected individuals [4], [6], [7].

Cospeciation studies of the genetic relationships between P-endosymbionts and their hosts usually reveal high levels of congruence. This suggests an ancient infection of an insect ancestor by a bacterium followed by its vertical transmission and subsequent cospeciation with the host [8]. In contrast, there is usually a lack of evolutionary congruence between the S-endosymbionts and their hosts, suggesting both multiple infections over time as well as horizontal transfer between unrelated hosts [4], [7], [9]–[13]. Despite the knowledge that S-endosymbionts can be transmitted horizontally, there is little data as to whether the level of horizontal transmission across the different S-endosymbionts is equivalent. One approach used to infer the transmission and evolutionary history of endosymbiont infections is to contrast the evolutionary relationships between the endosymbionts and their hosts.

Endosymbiont infections in arthropods are common [14] and amongst these, the whitefly, *Bemisia tabaci* (Gennadius) (Hemiptera: Aleyrodidae), contains the greatest known diversity with seven so far being described [8], [15], [16,]. Studies of the Aleyrodidae show that a distinct lineage within the gammaproteobacteria of P-endosymbiont is always present [17], [18] and has been given the provisional designation *Candidatus* ‘*Portiera aleyrodidarum*’ [19]. In addition, *B. tabaci* is also infected with several species of S-endosymbionts, *Candidatus* ‘*Hamiltonella defensa*’ (Enterobacteriaceae), *Arsenophonus*, *Cardinium* (Bacteroidetes), *Fritschea bemisiae* (Simkaniaceae), *Rickettsia* and *Wolbachia* [13], [16]–[18], [20]–[24]. Our knowledge of the function of these S-endosymbionts in *B. tabaci* is limited, but evidence suggests that within populations, the composition of S-endosymbionts varies both temporally and spatially [5], [8], [13], [16], [18], [20], [25]–[28]. Further, S-endosymbiont infections have been shown to carry a fitness benefit [24] as well as cost [29], [30].

Our most recent understanding of *B. tabaci* is that it is a cryptic species complex of at least 34 morphologically indistinguishable species [31]–[34] that exhibit complete or partial mating isolation [35]–[37]. In the case of complete mating isolation copulation does not usually occur whereas for partial mating isolation, the resulting fitness of the progeny is substantially reduced relative to the parents. The complex has a global distribution with a distinct geographic structure in terms of genetic relatedness, Sub-Saharan Africa, New World, Africa/Mediterranean/Middle East/Asia Minor, Indian Ocean/East Africa and Asia/Australia [31], [32], [38]. Two members of the complex, Middle East - Asia Minor 1 (MEAM1, commonly referred to as biotype B in the literature) and Mediterranean (MED, commonly referred to as biotype Q in the literature) have spread well beyond their respective home ranges through the trade in ornamental plants [38], [39] and both have invaded parts of Asia over the last 20 years [32]. As well as the invaders, Asia has at least 15 indigenous members of the complex; AsiaI, AsiaII_1, AsiaII_2, AsiaII_3, AsiaII_4, AsiaII_5, AsiaII_6, AsiaII_7, AsiaII_8, AsiaII_9, AsiaII_10, AsiaIII, China1, China2, China3 [31], [33]. The question therefore arises as to what is the cause of this level of species diversification. S-endosymbiont diversity is known to vary both within and between different members of the complex [5], [8], [13], [16], [18], [20], [25]–[28] and it is possible that they have contributed to the development of this diversity.

The presence of several cryptic species in the *B. tabaci* complex might be the result of reproductive isolation induced by S-endosymbionts (e.g. *Wolbachia*, *Arsenophonus*, *Cardinium*) [27], [40]–[42]. All S-endosymbionts are susceptible to horizontal transmission [11], [12], however the high prevalence of *Wolbachia* [14] across so many species that are unable to copulate suggests it may be particularly prone to horizontal transfer.

One approach to explore whether horizontal transfer is occurring is to compare the evolutionary relationships of the host with those of their endosymbionts. This is usually done using tree-based methods which compare the branching structure to determine whether tree topologies are more similar and if more codivergence events are present than would be expected by chance. Commonly used tree based methods include reconciliation analysis (TreeMap) [43] and generalized parsimony (TreeFitter) [44]. TreeMap is used to find optimal reconstructions of the history of association by maximizing cospeciation events and minimizing host shifts. It uses the Jungles analysis [45] so as to consider all potentially optimal solutions and so enables host shifts to be considered. TreeFitter assigns costs against the four types of co-phylogenetic events (cospeciation, duplication, sorting and host shift) [43], [46] and the optimal solution(s) is the one with the lowest global cost of reconstruction. Cospeciation is the joint speciation of two organisms with a close ecological association (usually a mutualistic or symbiotic relationship). In other words, cospeciation events occur when host and parasitoid species co-diverge and so display parallel cladogenesis. When incongruence occurs this signals the absence of cospeciation and instead suggests that host switching, sorting or duplication may be involved. Host switching events occur when a species successfully colonises a host species other than its current host. Duplication, or intrahost speciation, occurs when a species lineage diverges without the stimulus of host speciation and results in several closely related species on the descendant host lineage. Sorting events occur when species are entirely, or apparently, removed from host species. These reflect events where a species is predicted to occur, but does not [43], [47]. TreeMap and TreeFitter enable the null hypothesis that the two phylogenies are related randomly to be tested by comparing the scores of optimal reconstructions (number of cospeciation events for TreeMap and global cost for TreeFitter) with those of phylogenies that have been obtained randomly through the use of the permutation procedure. Because these programs require fully resolved trees, all combinations of the obtained tree topologies between the host and its endosymbionts need to be tested so as to account for phylogenetic uncertainty. TreeFitter also allows assignment of different costs to the four types of events and by varying these costs, the effect on the test results can then be compared. In addition to tree-based methods, host and parasitoid phylogenies can be assessed for similarity using distance-based and data based methods [48]–[53], but these latter methods are only valid when the genetic data being compared is homologous and as the data in this study are derived from different gene regions, only tree-based methods are appropriate [48], [54], [55]. This approach was therefore adopted to explore the evolutionary relationships between *B. tabaci*, its P-endosymbiont and three of its S-endosymbionts, *Arsenophonus*, *Cardinium* and *Wolbachia* and to so determine whether the patterns observed were more indicative of cospeciation and horizontal transfer events.

## Results

### Prevalence of endosymbionts among Asian *B. tabaci* cryptic species

All 570 individuals (30 individuals per location) collected from 19 different locations were infected with the species of primary endosymbiont, *P. aleyrodidarum* (Table S1). In case of the S-endosymbionts, 76.3% individuals of different *B. tabaci* cryptic species were positive for *Wolbachia*, 34.2% for *Arsenophonus* and 10.5% for *Cardinium* (Table S1). Out of a total of 19 collections, 16 positive collections were sequenced for primary endosymbionts and *Wolbachia*, ten for *Arsenophonus* and five for *Cardinium* based on *B. tabaci* cryptic species, geographical locations and plant hosts of *B. tabaci* cryptic species. Of the 16 different *Wolbachia* sequenced for the *wsp* gene, 11 were from members of the complex that are indigenous to Asia, two AsiaII_1, two AsiaII_7, five AsiaI and two China1 and five to invasive members of the complex, three MED and two MEAM1. For the *Wolbachia ftsZ* gene, 6 were from species indigenous to Asia, one China1, four Asia1, one AsiaII_7 and five that are invaders, three MED and two MEAM1 (Table S1). Of the 10 different *Arsenophonus* sequenced, three were from AsiaI, three from AsiaII_1, two from AsiaII_6, one from AsiaII_7 and one from MED while for the five *Cardinium*, three were from AsiaII_1, one from AsiaII_6 and one from MED (Table S1). All three individuals from each infected sample location were infected with the same haplotype of endosymbiont i.e. there was no variation within location.

### Phylogenetic analysis of host *B. tabaci* cryptic species and their endosymbionts

**Figure 1:**
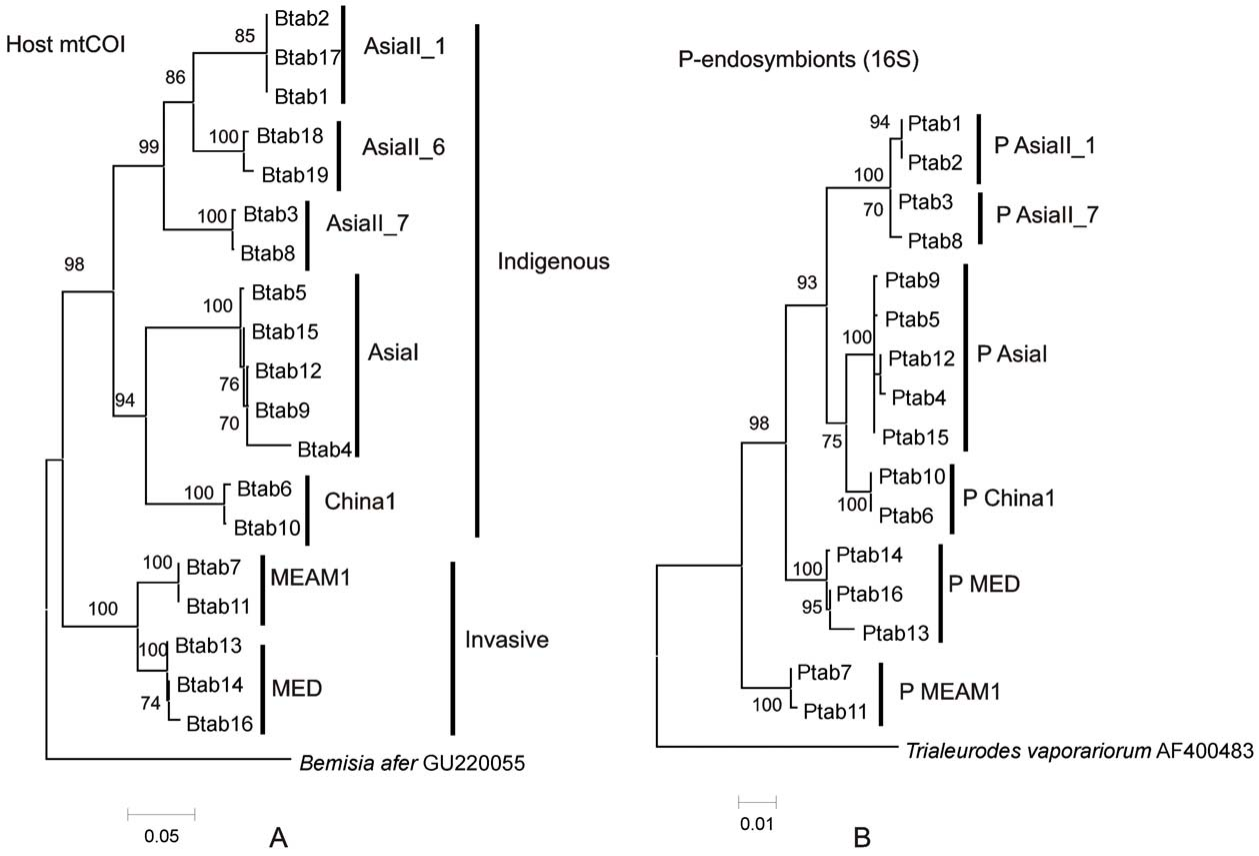
Phylogenies of the host *B. tabaci* cryptic species and their P-endosymbionts. A. Phylogenetic tree reconstruction based on mtCOI sequences (length = 830 bp) of host *B. tabaci* cryptic species using ML analysis under the HKY+G substitution model. The bootstrap values are indicated. *Bemisia afer* (GU 220055) is used as outgroup. Accession numbers for Btab1-19 submitted to GenBank are JX428681–JX428696. All mtCOI sequences of host *B. tabaci* cryptic species used in this study were clustered with other blast references sequences from GenBank and their ML phylogenetic reconstruction is shown Fig. S1A. B. Phylogenetic tree reconstruction based on 16S rRNA gene sequences (length = 1100 bp) of P-endosymbionts of *B. tabaci* cryptic species using ML analysis under the HKY+G substitution model. The bootstrap values are indicated. *Trialeurodes vaporariorum* (AF400483) is used as the outgroup. Accession numbers for Ptab1-Ptab16 submitted to GenBank are JX428713–JX428731. All 16S sequences of P-endosymbionts used in this study were clustered with other blast references sequences from GenBank and their ML phylogenetic reconstruction is shown Fig.. S1B.

The phylogenetic reconstruction for *B. tabaci* complex from Asia and the clusters assigned to each of the different species is shown in Fig. 1A and is consistent with previously published trees [31], [32]. The relationship between the different *P. aleyrodidarum* is shown in Fig. 1B. The relationships for the most part mirror those of *B. tabaci* (Fig. 1B).

**Figure 2:**
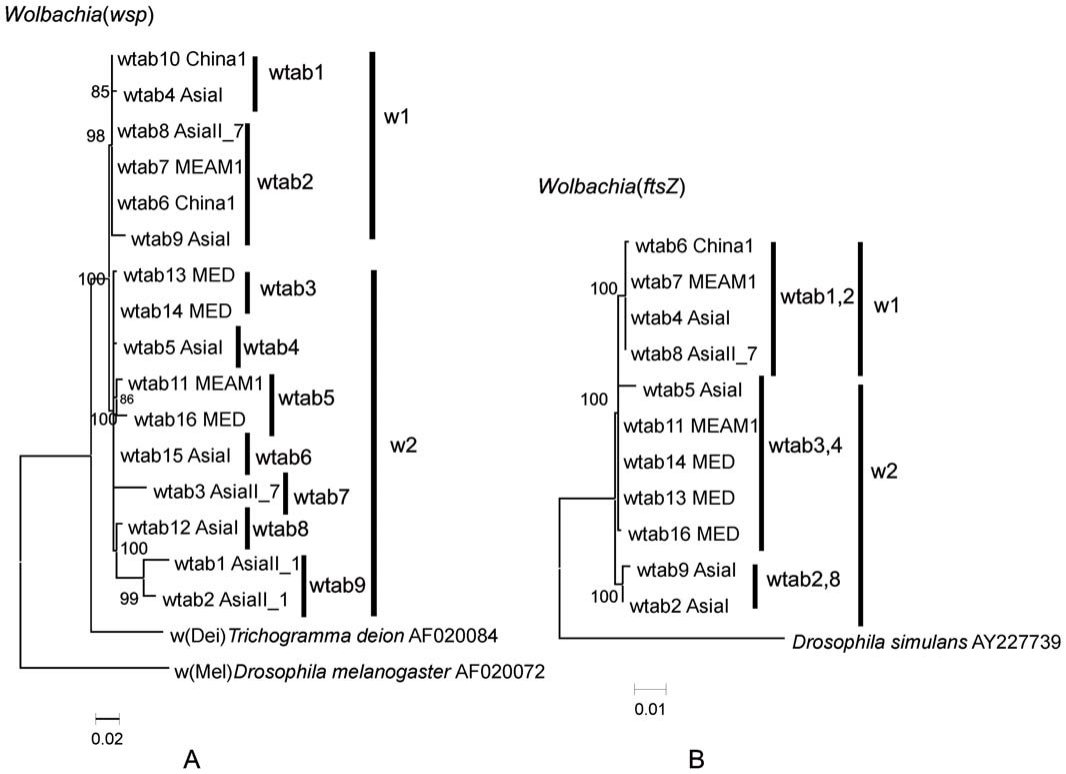
Phylogenies of the S-endosymbiont *Wolbachia*. A. Phylogenetic tree reconstruction based on *wsp* gene sequences (length = 480 bp) of *Wolbachia* of *B. tabaci* cryptic species using maximum-likelihood analysis under the T92 substitution model. The bootstrap values are indicated. *Trichogramma deion* (AF020084) and *Drosophila melanogaster* (AF020072) are used as outgroups. Accession numbers for wtab1-16 submitted to GenBank are JX428697–JX428712. All *wsp* sequences of *Wolbachia* used in this study were clustered with other blast references sequences from GenBank and their ML phylogenetic reconstruction is shown Fig. C. B. Phylogenetic tree reconstruction based on *ftsZ* gene sequences (length = 850) of *Wolbachia* of *B. tabaci* cryptic species using ML analysis under the TN93 substitution model. The bootstrap values are indicated. *Drosophila simulans* in case of *ftsZ* gene (AY227739) is used as the outgroup. Accession numbers for sequences used in the tree are JX428732–JX428742. All *ftsZ* sequences of *Wolbachia* used in this study were clustered with other blast references sequences from GenBank and their ML phylogenetic reconstruction is shown Fig. S1D.

The 16 *Wolbachia* were grouped into two main clusters, W1 and W2 (Fig. 2 A, B). All *Wolbachia* sequences found in this study belonged to supergroup B (Fig. 2 A, B). The relationships between different *Wolbachia* based on their *wsp* and *ftsZ* sequences are shown in Fig. 2 A and B, respectively. The *wsp* tree has nine well supported clades (bootstraps >70%), wtab1-wtab9. Clades wtab1, 4, 6–9 consist of *Wolbachia* from indigenous Asian members of the complex only and wtab3 and wtab5 consist of invader members only whereas wtab2 has *Wolbachia* from both indigenous Asian and invader members of the *B. tabaci* complex (Fig. 2 A).

The *ftsZ* tree has three well supported clades (bootstraps >70%). The *Wolbachia* from different clades of *wsp* tree were clustered together in same clade here in *ftsZ* tree (Fig. 2 A). Out of three clades of *ftsZ* tree, the top clade consists of *Wolbachia* from indigenous Asian members as well as one from invader member of the complex whereas the middle clade consists of *Wolbachia* from invader members along with one from indigenous member and the bottom clade consists of *Wolbachia* from only indigenous Asian members of the complex (Fig. 2 A).

**Figure 3:**
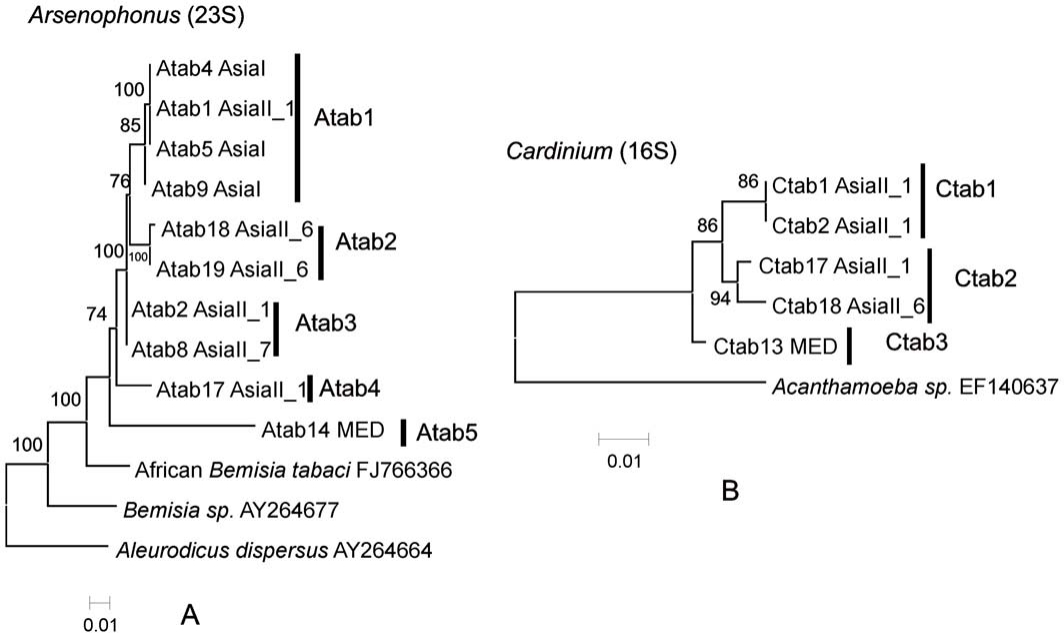
Phylogenies of the S-endosymbionts *Arsenophonus* and *Cardinium*. A. Phylogenetic tree reconstruction based on 23S rRNA gene sequences (length = 550 bp) of *Arsenophonus* of *B. tabaci* cryptic species using ML analysis under the HKY+G substitution model. The bootstrap values are indicated. African *B. tabaci* (FJ66366), *Bemisia sp.* (AY264677) and *Aleurodicus disperses* (AY264664) are used as the outgroups. Accession numbers for sequences used in the tree are JX428666–JX428675. All 23S sequences of *Arsenophonus* used in this study were clustered with other blast references sequences from GenBank and their ML phylogenetic reconstruction is shown Fig. S1E. B. Phylogenetic tree reconstruction based on 16S rRNA gene sequences (length = 400) of *Cardinium* of *B. tabaci* cryptic species using MLd analysis under the K2 substitution model. The bootstrap values are indicated. *Acanthamoeba sp.* (EF140637) is used as the out group. Accession numbers for sequences used in the tree are JX428676–JX428680. All 16S sequences of *Cardinium* used in this study were clustered with other blast references sequences from GenBank and their ML phylogenetic reconstruction is shown Fig. S1F.

All the *Arsenophonus* grouped into two well supported clusters Atab1-Atab5 (Fig. 3A). Atab5 had a single representative from MED whereas Atab1-Atab4 represented individuals found in indigenous Asian *B. tabaci* cryptic species.

Each of the five different *Cardinium* was belonging to one of two clusters, one containing two groups Ctab1 and Ctab2 and the other Ctab3. Ctab3 was from the invasive MED whereas the others represented individuals from indigenous Asian *B. tabaci* cryptic species.

### Co-phylogenies of *B. tabaci* host and its endosymbionts

**Figure 4:**
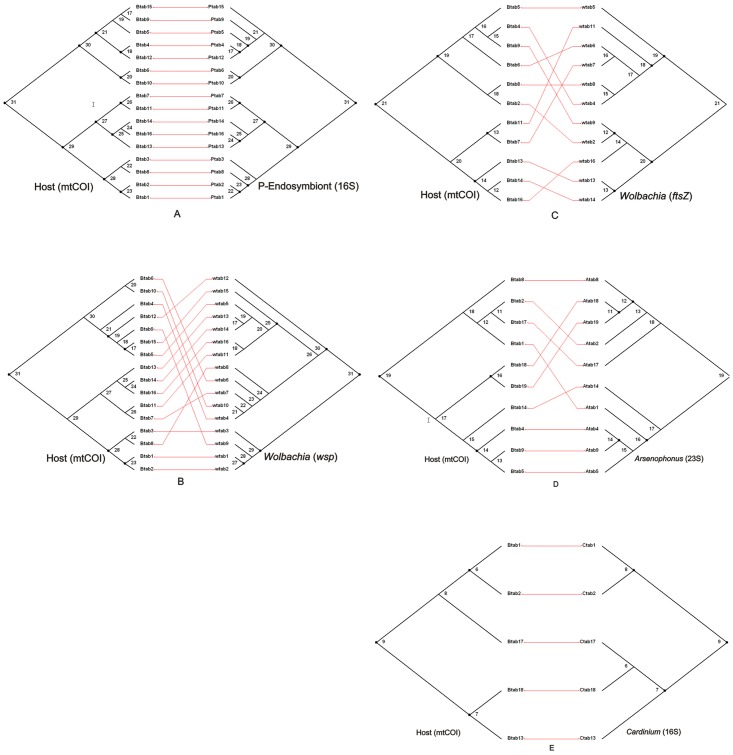
Comparisons of *B. tabaci* cryptic species and endosymbionts ML phylogenies. Black dots show cospeciation points. **A.** Host *B. tabaci* mtCOI versus P-endosymbiont, *P. aleyrodidarum* 16S rRNA gene. **B.** Host *B. tabaci* mtCOI versus S-endosymbiont, *Wolbachia wsp*. **C.** Host *B. tabaci* mtCOI versus S-endosymbiont, *Wolbachia ftsZ* genes. **D.** Host *B. tabaci* mtCOI versus S-endosymbionts, *Arsenophonus* 23S rRNA gene. **E.** Host *B. tabaci* mtCOI versus S-endosymbionts, *Cardinium* 16S rRNA gene.

There was high congruence between the *B. tabaci* mtCOI and *P. aleyrodidarum* 16S rRNA gene phylogenies (Fig. 4A). In contrast, there is less agreement between the *B. tabaci* mtCOI and the S-endosymbiont phylogenies. The *Wolbachia* and *B. tabaci* phylogenies were incongruent (Fig. 4B, C) whereas the phylogenies for *Cardinium* and *Arsenophonus* showed partially congruence with *B. tabaci* (Fig. 4D and E).

Analysis of the single optimal topology for mtCOI with the ML and MP topologies for endosymbionts using TreeFitter at a *p-*value significance cut-off of 0.05 showed 12 cospeciation events and only two host switching and sorting events between *B. tabaci* and their P-endosymbionts (Fig. S2A). The two observed events of host switching in P-endosymbionts were actually between populations of same species (Fig. S2A). In the case of *Wolbachia* while there was evidence to support six to eight cospeciation events (six in the case of *ftsZ* and eight in the case of *wsp*), there was evidence to support a considerable number of random host switches (3–7) and sorting events (13–14) (Fig. S2 B, C). This was also the case for *Arsenophonus*, five cospeciation events, four host switches and four sorting events (Fig. S2D) and for *Cardinium*, three cospeciation events, one host switch and one sorting events (Fig. S2E).

### Geographical correlations of *B. tabaci* cryptic species and its endosymbionts

There were no significant correlations between host genetic variation and geographical distances in the case of both invasive and indigenous cryptic species and their P-endosymbionts (r = −0.23, P = 0.097 for indigenous; r = −0.112, P = 0.80 for invasive). Likewise, there are no significant correlations between S-endosymbionts genetic variation (measured separately from invasive and indigenous host cryptic species) and geographical distances between the collection site in case of *Wolbachia* using *ftsZ* gene (r = −0.072 P = 0.081 for indigenous; r = 0.232 P = 0.60 for invasive) or using *wsp* gene (r = −0.27, P = 0.197 for indigenous; r  = 0.203, P = 0.66 for invasive) as well as in case of *Arsenophonus* (r = 0.083,P = 0.75 for indigenous) or *Cardinium* (r = −0.01, P = 1.00 for indigenous) suggesting that their genotypes are not geographically restricted.

### Host mtCOI correlation with P- and S-endosymbionts

The P-endosymbiont, *Wolbachia* and *Arsenophonus* were significantly correlated with host mtCOI (Table 1). The correlation was highest for the P-endosymbiont followed by *Arsenophonus* then *Wolbachia* (Table 1). The small dataset for *Cardinium* lead to no significant correlation (Table 2). *Wolbachia* (*wsp* gene) was least, but with significant correlation with host genetic diversities while *Wolbachia* (*fstZ* gene) was not correlated with host genetic variations (Table 1).

**Table 1:** The mantel correlations between the genetic differences between *B. tabaci* sequences and that of their endosymbionts.

Endosymbionts Genes	n	Lower 95% CI	correlation	Upper 95% CI	*P*
P-Endosymbionts (16S rRNA)	16	0.75	0.78	0.83	0.0001
Arsenophonus (23S rRNA)	10	0.36	0.50	0.60	0.0001
*Wolbachia* (*wsp*)	16	0.10	0.17	0.26	0.0156
*Wolbachia (fstZ)*	11	−0.30	−0.025	0.26	0.8531
Cardinium (16S rRNA)	5	−0.30	0.33	0.82	0.3610

Not all samples had all the endosymbionts resulting in different sample sizes (n). The lower and upper 95% confidence intervals (CI) of estimates are also given.

**Table 2:** Summary of co-phylogenetic comparisons of host species and their endosymbionts ML phylogenies.

Endosymbionts Genes	Cospeciation	Duplication	Host switch	Sorting
P-Endosymbionts (16S rRNA)	12	0	2	2
*Arsenophonus* (23S rRNA)	5	0	4	4
*Wolbachia* (*wsp*)	8	0	7	13
*Wolbachia* (*fstZ*)	6	1	3	14
Cardinium (16S rRNA)	3	0	1	1

The reconstruction shows the co-phylogenetic analysis between the mtCOI gene of *B. tabaci* cryptic species with the genes of their primary endosymbionts and secondary endosymbionts.

Overall, both distance and tree based analyses produced a very similar result. In the tree based analysis, there was high cospeciation in the case of P-endosymbionts as compared to S-endosymbionts and there are more host switches for the S-endosymbionts, especially in *Wolbachia* (Table 2, Fig. 4). Similarly in the distance based analysis, P-endosymbionts are highly correlated with their host as compared to S-endosymbionts with *Wolbachia* showing the lowest level of correlation (Table 1).

## Discussion

The results confirmed a very high degree of co-cladogenesis between *B. tabaci* and *P. aleyrodidarum*; this was expected and has been shown in numerous studies [56]–[58] and suggests a single infection of a whitefly ancestor with a bacterium followed by vertical transmission to the progeny. In contrast, the phylogenies of the S-endosymbionts were largely incongruent with that of the host. One explanation is that P-endosymbionts are always found within the bacteriocytes [4] which are highly specific cells with a limited distribution within the host. This suggests that being limited to the bacteriocytes makes it more difficult for horizontal transmission and this is supported by the very low number of host switches and sorting events. In contrast, S-endosymbionts are not limited to the bacteriocytes [4], [6] and the much smaller number of cospeciation events and much higher numbers of host switches and sorting events suggest that being able to reside in multiple cell types makes it easier for horizontal transmission to occur. Of the S-endosymbionts, *Wolbachia* showed a lack of congruence whereas *Arsenophonus* and *Cardinium* showed partial congruence. This suggests that of these S-endosymbionts, *Wolbachia* may be particularly adapted to undergoing horizontal transmission [11], [12]. An alternative explanation is that P-endosymbionts are essential and so there may be extremely strong selection on both host and bacterium to maintain the association [2]. In contrast, S-endosymbionts are either parasites or mutualists. In the case of the former, the association is likely to be unstable due to selection for host resistance while in the later, the association is subject to changes in ecological circumstance and so lost when environmental change selects against the association [7], [59].

It is generally assumed that vertical transmission of *Wolbachia* predominates within species and that horizontal transfer is a rare event [60], [61]. As a consequence, one would expect the association between the *Wolbachia* genome and the mitochondrial genome to be non-random i.e. in linkage disequilibrium as both are vertically transmitted from mother to offspring [62]. However, if horizontal transmission is occurring then one would expect the relationship between the two genomes to become decoupled; the extent of which would be indicated by the level of incongruence between the two phylogenies. Our results show a considerable degree of congruence suggesting that horizontal transmission was occurring at a rate sufficient to mask linkage disequilibrium.

The lack of congruence between mitochondrial and *Wolbachia* gene genealogies cannot be explained in terms of a single infection event followed by co-divergence within the species. Instead, it is strongly compatible with extensive shuffling within the species. Overall, based on the *Wolbachia*/mtCOI data, the pattern that emerges is a complex history of infection in *B. tabaci* that is shaped by both vertical and horizontal transmission plus frequent turnover (loss and replacement) within a single species. The higher infection frequency in *B. tabaci* and the common occurrence of multiple infections in *B. tabaci* are good arguments that horizontal transfers occurs between different cryptic species of *B. tabaci*. High rates of horizontal transmission are one explanation for the frequency of *Wolbachia* infections that have been observed both across the *B. tabaci* complex as well as in arthropods in general [13], [14], [47]. Moreover, *B. tabaci* has been shown to harbor different *Wolbachia* [13]. Theoretically, it has been shown that two different *Wolbachia* strains can coexist stably in parapatric host populations [63] and that bidirectional cytoplasmic incompatibility reduces the gene flow of locally adapted alleles and selects for pre-mating isolation [63], [64]. Research indicates that different *Wolbachia* may contribute to reproductive isolation through cytoplasmic incompatibility [65]. It may therefore be that cytoplasmic incompatibility in combination with relatively high rates of horizontal transmission has been promoting the high level of diversity observed across the *B. tabaci* cryptic species complex. In addition to *Wolbachia*, *Cardinium* and *Arsenophonus* are also known to manipulate host reproduction in a wide range of insect species including *B. tabaci* [27], [59].

Extensive collections of MEAM1 across its home range (Middle East and Asia Minor) and invaded range have not provided any good evidence that the invading MEAM1 was infected with *Wolbachia* prior to its invasion of China [8], [15], [16], [31], [32], [38], [66]. There is one study [67] that identifies MEAM1 infected with *Wolbachia* in Israel, but this observation is in doubt as extensive sampling across Israel has since been unable to find any evidence for *Wolbachia* infections in MEAM1 (Zchori Fein personnel communication). MEAM1 most likely invaded China in the mid-1990s and studies investigating the presence of S-endosymbiont infections in MEAM1 in mainland China reported no presence of *Wolbachia* at least up to 2005 whereas studies from 2007 onwards have reported varying levels of infections with *Wolbachia* [13], [18], [28], [66]–[68, Ahmed MZ et al. unpublished data]. The presence of similar *Wolbachia* in both Asian indigenous (AsiaI and China1) and exotic members of the *B. tabaci* complex provides good support for the argument that the *Wolbachia* in MEAM1 in China was acquired from the indigenous Asian members of the complex and that this has occurred within the last 6 years. On the other hand, the other exotic invader found in China, MED, was first detected in China in 2003 [69]. MED was known to have been infected with *Wolbachia* before its invasion of China [13], [18] and our study shows a close association between the *Wolbachia* from Chinese MED and that found in the Mediterranean home range. The observation that some of the *Wolbachia* infecting AsiaI in China are less related to those from other indigenous Asian *Wolbachia* and more related to *Wolbachia* of Mediterranean origin suggests that there has been a recent horizontal transfer of *Wolbachia* from the invading MED to the indigenous AsiaI and that this has occurred since 2003.

Our study showed that the indigenous Asia1 also harbored another *Wolbachia* infection that was the same as the one found in MEAM1. However, this also occurred in the other *B. tabaci* species to have invaded China, MED. This also occurs in MED from Egypt and suggests a second horizontal transfer event in China from MED to Asia1 as well as MEAM1 and again this transfer has occurred within the last 10 years.

Intra- and interspecific horizontal transfers of *Wolbachia* occur between organisms that interact in close confinement [70]. In general, intraspecific horizontal transfer is more successful than interspecific transfer [70] and is most likely due to incompatibilities between *Wolbachia* and hosts' nuclear/cytoplasmic background [12], [71]. The apparently frequent interspecific horizontal transmission between different members of the *B. tabaci* complex suggests that there is sufficient similarity between the different members of the complex to support the *Wolbachia* from related members of the complex. This is supported by the numerous observations that have been made in regards to courtship interactions between different members of the complex [72]. While copulation seldom, if ever, occurs, there is still a complex courtship process which suggests that the different species are still sufficiently close that interspecies mate recognition persists.

The observation of a number of apparent horizontal transfer events between different members of the *B. tabaci* complex raises the question as to the mechanism that may be enabling this to occur. The horizontal transfer of *Wolbachia* from an infected whitefly to other insects as a result of feeding on the same leaf substrate has been suggested as one mechanism [73]. For this to be possible, the bacterium needs to be small enough to pass through the salivary duct. Recent research involving mosquitoes suggests that the bacterium may be too large to pass through the salivary duct making the prospect of transmission through the plant unlikely [74].

Another possible route for horizontal transmission is parasitoids, of which numerous species parasitise the different members of the *B. tabaci* complex [75]. One study has shown that *Rickettsia* from *B. tabaci* were readily able to infect the larvae of two species of parasitoid within the whitefly host, but were unable to be vertically transmitted as the bacteria were unable to infect the parasitoid's oocytes [76]. Gueguen et al. [15] also found evidence for horizontal transfer between different members of the *B. tabaci* complex and suggested parasitoids may be involved. In contrast, it has been argued that transfer from parasitoids to hosts was quite unlikely [12], but as discussed above, the systems being considering did not involve closely related hosts and so the cytoplasmic backgrounds were likely to be quite different.

It has also been suggested that endosymbiont communities can be used to resolve taxonomic distinction within the *B. tabaci* complex [15], [20], [77]. As has been shown in aphids [78], our results indicate that the P-endosymbionts may be a useful means of considering the taxonomic relationships within the *B. tabaci* complex. However, the low level genetic linkage between the host and their S-endosymbionts, especially *Wolbachia*, due to frequent host shifts through horizontal transfer suggest that they have little role to play in resolving the taxonomy of the *B. tabaci* complex.

## Conclusion

Endosymbionts of *B. tabaci* cryptic species show evidence for variable degrees of horizontal transmission. Primary endosymbionts are almost entirely vertically transmitted whereas the secondary endosymbionts and especially *Wolbachia*, show evidence of horizontal transfers between different species within the *B. tabaci* species complex. This also suggests that secondary endosymbiont genetic variation may not reflect host genetic variation and should not be used to infer taxonomic relationships within the host species complex. This is not surprising as secondary endosymbionts are facultative in their association with the host and so not always present within all individuals within a species. *Wolbachia* shows evidence a number of horizontal host shifts across the complex with three occurring within the last 20 years. This suggests that it may move readily between different members of the complex and most likely via parasitoid activity. If this is the case, then it is possible that *Wolbachia* may be playing a role in driving the process of speciation with the *B. tabaci* species complex and may help explain the high level of species level diversity that is being increasingly observed.

## Materials and Methods

### Whiteflies sampling

*Bemisia tabaci* were collected from 19 locations in China between 2007 and 2010. All collections were undertaken by the Department of Entomology, South China Agricultural University, China and no any specific permission was required to collect any of these samples. The identity of the species was determined by comparing their mitochondrial cytochrome oxidase one (mtCOI) against published consensus sequences [31] and using the assignment rules from that study [31], [79].

### DNA extraction and PCR amplification

DNA was extracted from individual adult females using a previously published method [13], [18]. The mtCOI was amplified using the primer pair C1-J-2195/TL2-N-3014 [80]. The *Portiera* 16S ribosomal RNA gene was amplified using the 28F and 1098R primer pair [8] while *Wolbachia wsp* was amplified using the wsp81F and wsp691R primers [10] and *ftsZ* using *Wolbachia* specific primers [9], the *Cardinium* 16S rRNA gene was amplified by Ch-F and Ch-R primers [81] and the *Arsenophonus* 23S rRNA gene was amplified using Ars23SF and Ars23R [23]. Amplification for the respective primer sets followed previously published methods [10], [23], [80], [81].

### Sequence alignments, measurement of genetic distances and phylogenetic analysis

Several recent studies have shown that exchange of genetic information can occur among *Wolbachia* supergroups [82], [83] and/or among strains within the same individual [84]. It has been demonstrated that recombination occurs across the whole *Wolbachia* genome [83] and in most of genes including *wsp*, *gltA*, *dnaA*, *ftsZ* and *groEL* [83], [85], [86]. As a result the two of the most frequently used genes (*wsp* and *ftsZ*) were used for genotyping *Wolbachia*, but because of the concern that recombination might influence the patterns observed, recombination analyses were undertaken to assess the level of recombination and identify portions of amplified fragment where recombination had taken place. All amplified gene products were cloned and three clones then sequenced. An additional three clones were sequenced if any sequence variation was detected in the first three clones. The sequences were first analyzed and aligned with DNAStar (Lasergene V5.0) and ClustalX 1.83 [87]. Genetic distances were calculated using the Kimura 2-parameter model in MEGA [88]. The phylogenetic relationships among the different cryptic species and their endosymbionts were analyzed by Maximum Likelihood (ML) using MEGA5 and PAUP*4.0b10 [88], [89]. The tree is drawn to scale, and branch lengths are measured in number of substitutions per site using MEGA5. Bootstrap values >70% from 1,000 iterations are shown.

### Recombination analysis

The consequences of incorrect phylogenetic signals caused by recombination can be avoided by first detecting recombination events prior to phylogenetic analysis. Two methods were used to consider recombination. The first was the bootscan method, implemented in the program Simplot [90] to construct replicate trees in a sliding window approach. The threshold level of 70% of the permutated trees in a given window for the detection of a recombination was used. The second used a non-phylogenetic method to apply dynamic programming to minimize the mutation and recombination cost between the various sequences used. This method was implemented in the software RECCO [91] which tests the hypothesis of no recombination and in line with Maydt and Lengauer [91] a significance level of 0.05 as the cutoff was used. The number of permutations was set at 1000, gap extension cost to 0.2, max α for the permutation test to 1 and the significance level to P≤0.05. The parameter α, representing the ratio of mutation cost to recombination cost was set to 0.2, the methods used to calculate mutation and recombination costs were respectively, Hamming and Delta Dirac. Those sequences where significant recombination was identified were not used in the subsequent phylogenetic analyses. Moreover *wsp* sequences have a number of hypervariable regions which on the basis of Braig et al. [92] and Nirgianaki et al. [20] were excluded from the analysis; this reduced the number of bases available for analysis from 625 to 480.

### Co-phylogenetic analysis

The Mantel test on the genetic distance matrices of cospeciating hosts and parasitoids was used to test for significant association between the matrices [48]. TreeFitter was then used to compare the single optimal topology derived from the combined mtCOI sequences of each *B. tabaci* species and each of ML and MP topologies derived from the combined gene sequences of P- and S-endosymbionts. All tests were performed based on 999 permutations. The support for cospeciation, host-switching, lineage sorting and duplication was explored using the exact search and best reconstructions option in TreeMap 2.0b. Only branches in the ML tree that were supported by at least 85% bootstrap support were considered [93].

### Correlation analysis

To test whether the genetic differences between *B. tabaci* increased with geographic distance, Mantel correlations between *B. tabaci* genetic differences and mapped distances between collections were undertaken using the mantel function in the ecodist package [94] in R version 2.13.0 [R Development Core Team 2011]. The same approach was then used to analyze how well endosymbiont genetic variability correlated with differences in whitefly genetic variability. Geographical distances between the locations from where the whiteflies were collected were calculated using their GPS coordinates.

## Supporting Information

Figure S1 **Phylogenetic tree reconstruction of** ***B. tabaci*** **and its endosmbionts based on various gene sequences.** **A.** Phylogenetic tree reconstruction based on mtCOI gene sequences (length = 830 bp) of host *B. tabaci* cryptic species using maximum-likelihood analysis under the HKY+G substitution model. The bootstrap values are indicated. **B.** Phylogenetic tree reconstruction based on 16S rRNA gene sequences (length = 1100 bp) of P-endosymbionts of *B. tabaci* cryptic species using maximum-likelihood analysis under the JC+G substitution model. The bootstrap values are indicated. **C.** Phylogenetic tree reconstruction based on *wsp* gene sequences (length = 480 bp) of *Wolbachia* of *B. tabaci* cryptic species using maximum-likelihood analysis under the T92+G substitution model. The bootstrap values are indicated. **D.** Phylogenetic tree reconstruction based on *ftsZ* gene sequences (length = 850) of *Wolbachia* of *B. tabaci* cryptic species using maximum-likelihood analysis under the TN93+G+I substitution model. **E.** Phylogenetic tree reconstruction based on 23S rRNA gene sequences (length = 550 bp) of *Arsenophonus* of *B. tabaci* cryptic species using maximum-likelihood analysis under the HKY+G substitution model. The bootstrap values are indicated. **F.** Phylogenetic tree reconstruction based on 16S rRNA gene sequences (length = 400) of *Cardinium* of *B. tabaci* cryptic species using maximum-likelihood analysis under the K2+G substitution model. The bootstrap values are indicated.
(DOC)

Figure S2 **The exact search at best reconstructions using TreeMap 2.0b show the ML phylogenetic comparison of endosymbionts over their hosts.** Endosymbionts displayed in black and the hosts in light grey; in reconstruction; cospeciation events are shown by (•), duplications by(▪) and host switches by (→). **A.** Host *B. tabaci* and its P-endosymbionts. **B.** Host *B. tabaci* and its *Wolbachia* (*wsp*). **C.** Host *B. tabaci* and its *Wolbachia* (*ftsZ*). **D.** Host *B. tabaci* and its *Arsenophonus* (23S). **E.** Host *B. tabaci* and its *Cardinium* (16S).
(DOC)

Table S1 **Asian** ***B. tabaci*** **cryptic species population sampled for S-endosymbionts.**
(DOC)
