# Supplementary figures and images for: Evidence for Horizontal Transmission of Secondary Endosymbionts in the Bemisia tabaci Cryptic Species Complex
Source: PLoS One. 2013 Jan 7;8(1):e53084. doi: 10.1371/journal.pone.0053084 (PMC3538644; doi:10.1371/journal.pone.0053084)

**Fig. S1A**


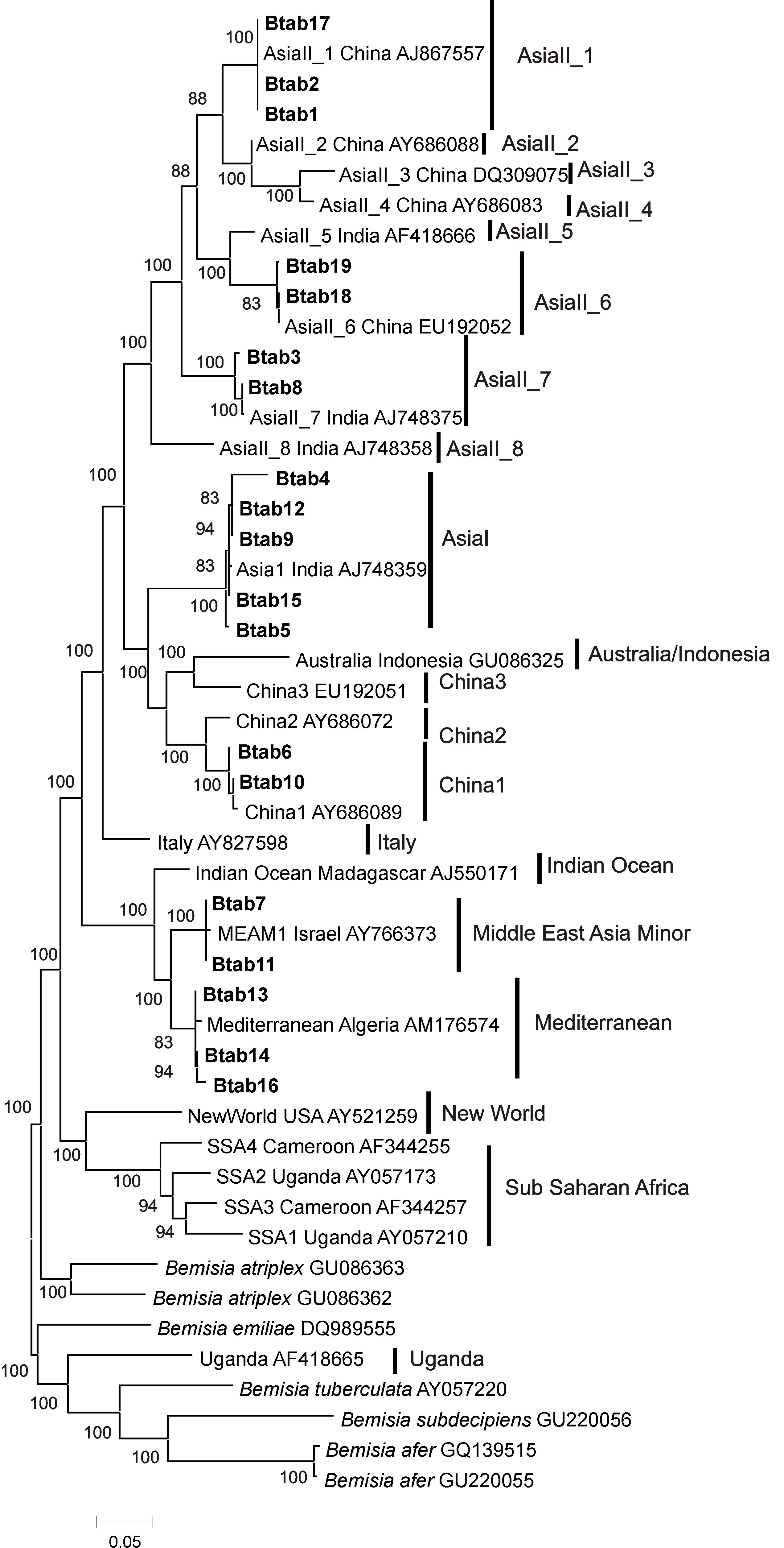


**Fig. S1B**


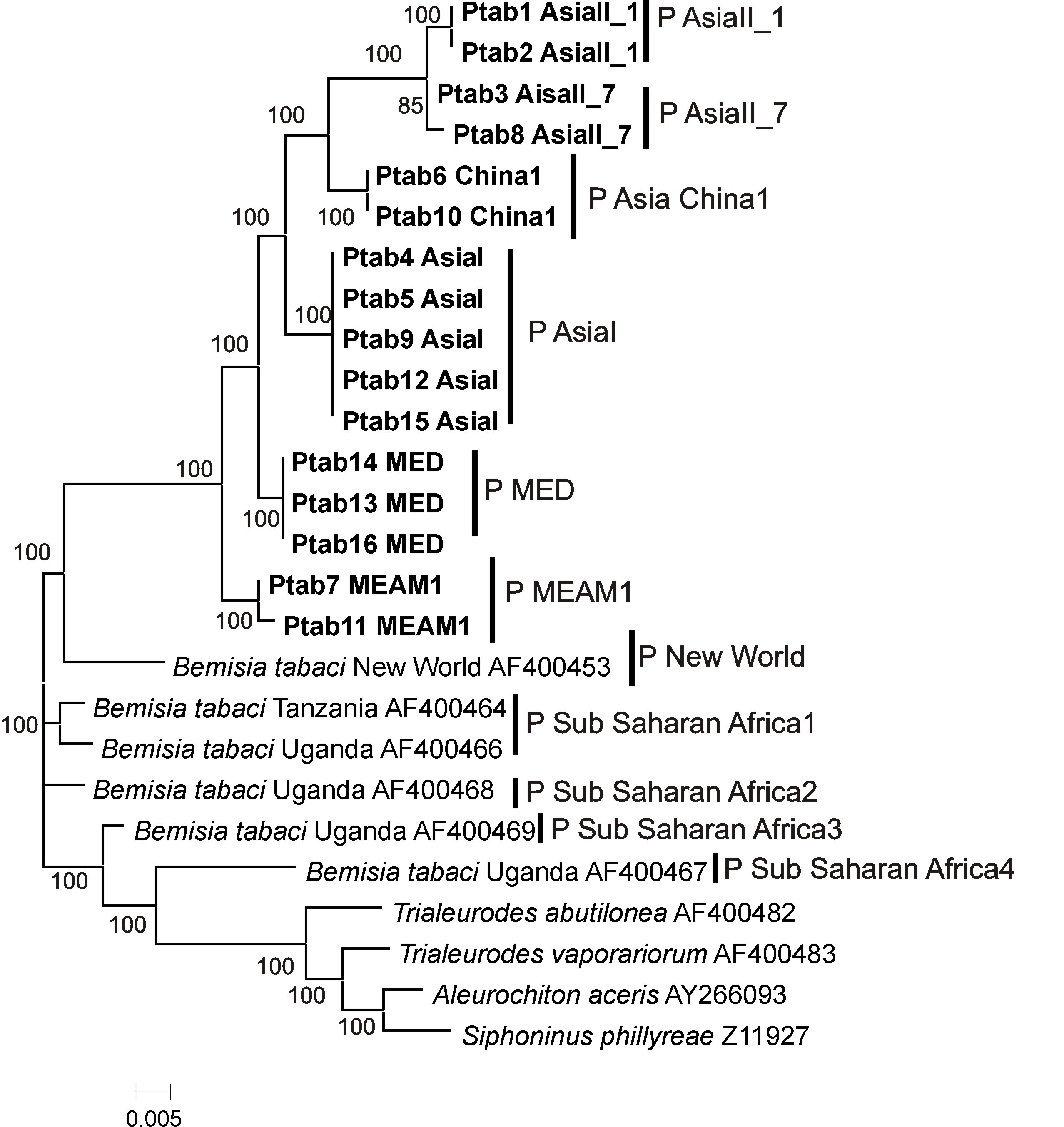


**A**


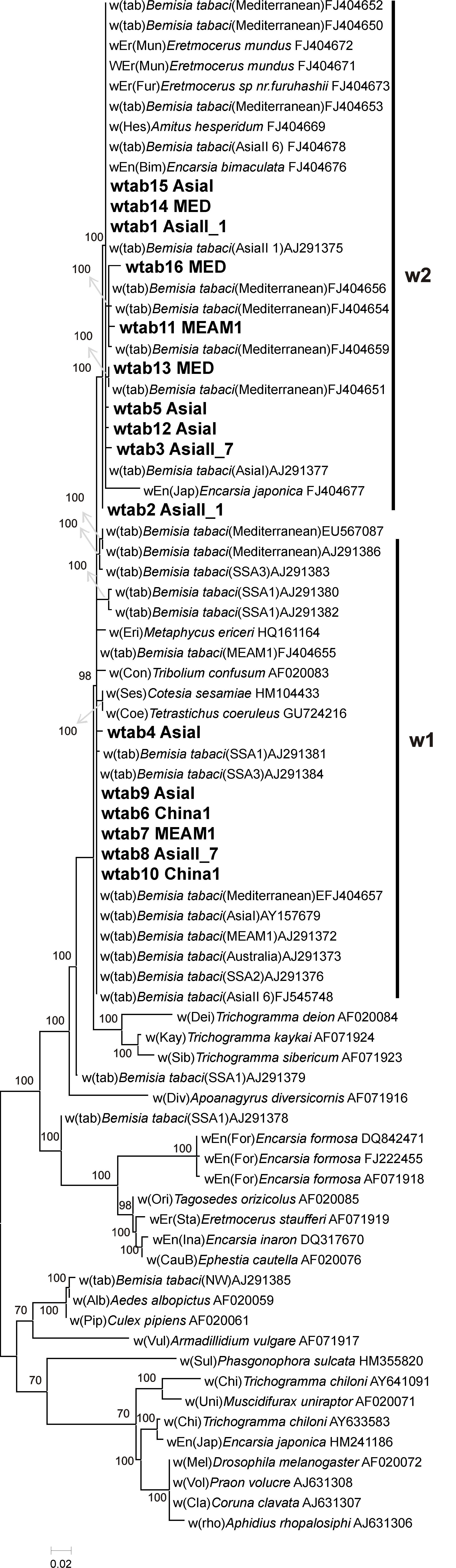


**Fig. S1C**


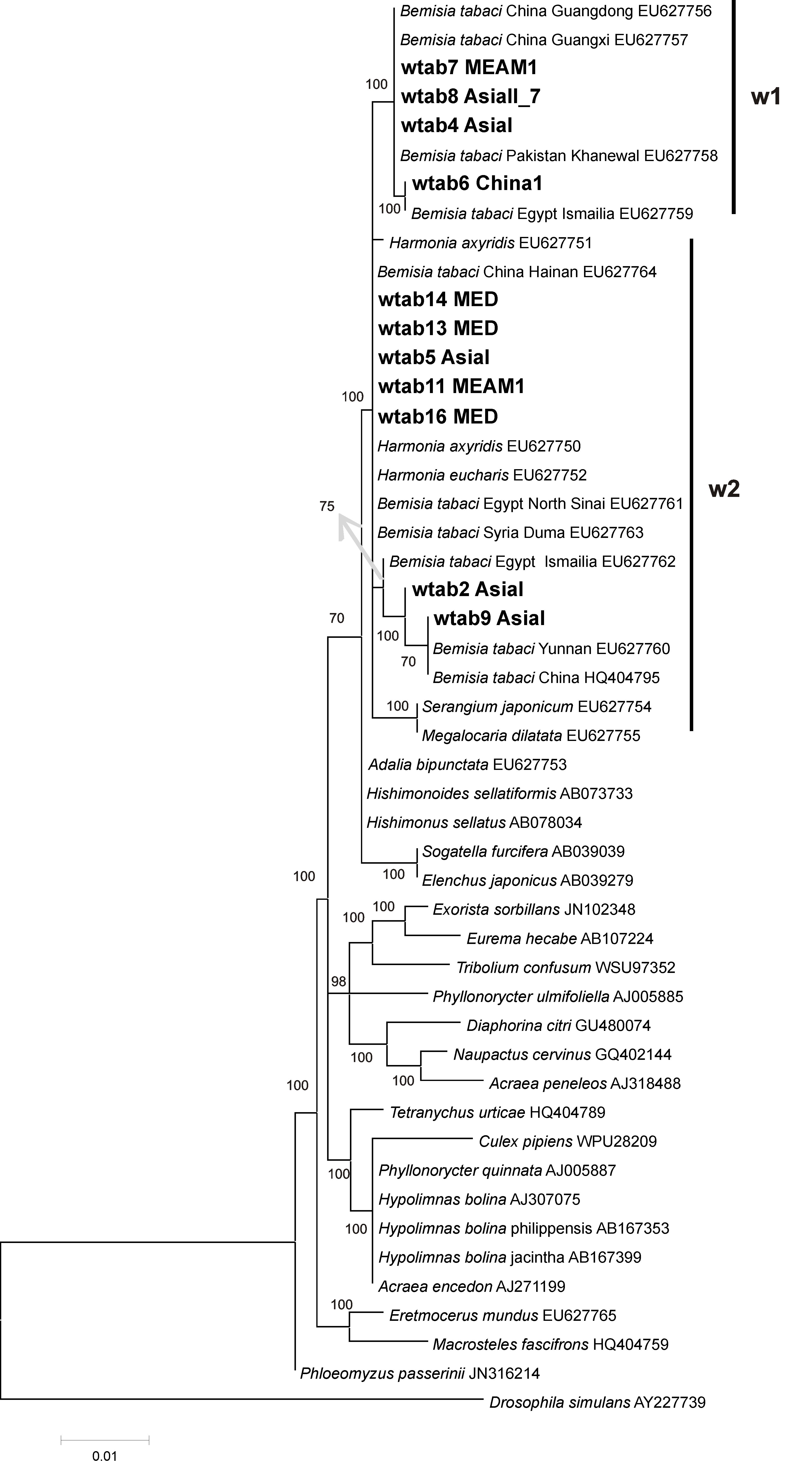


**Fig. S1D**

**
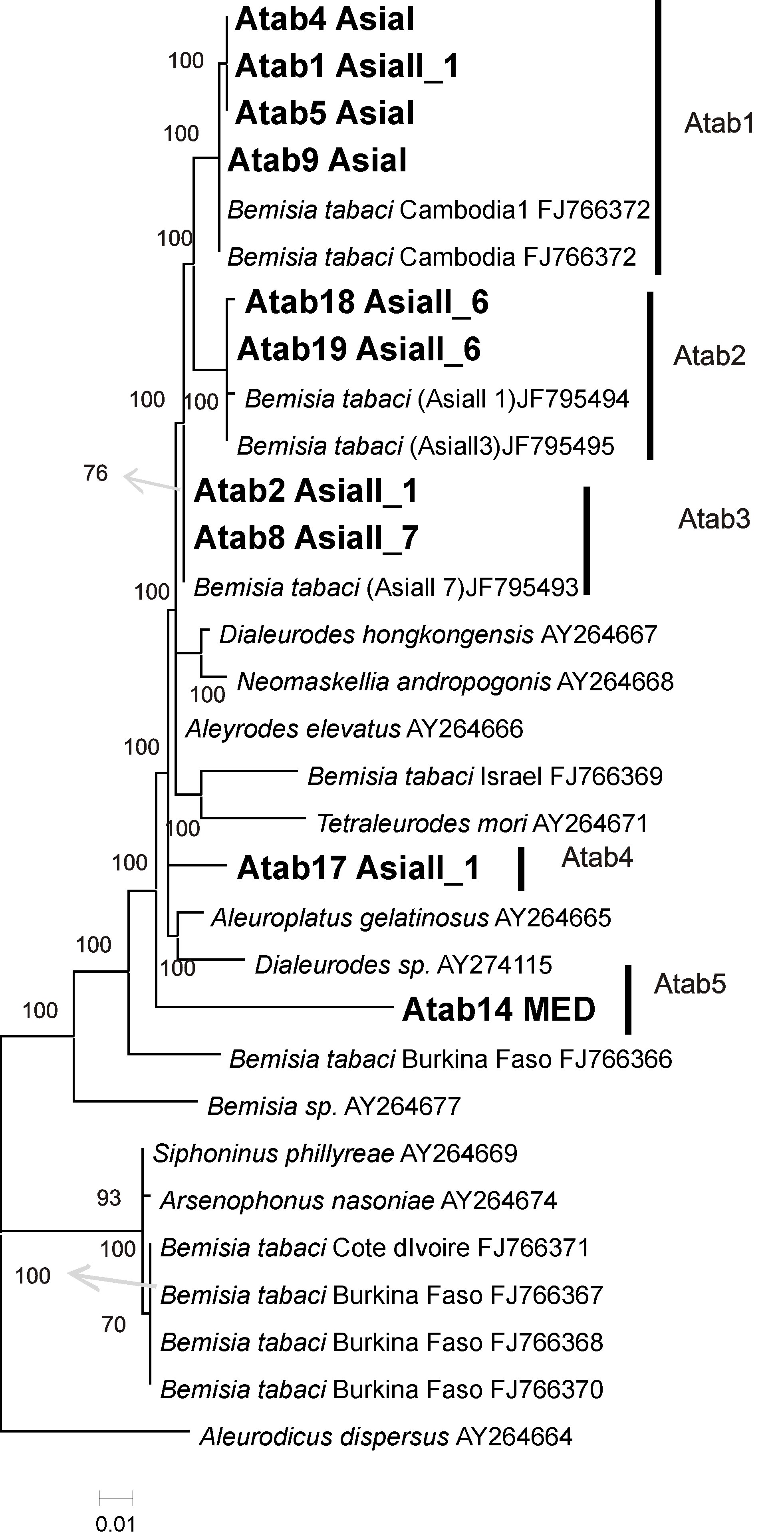
**

**Fig. S1E**

**
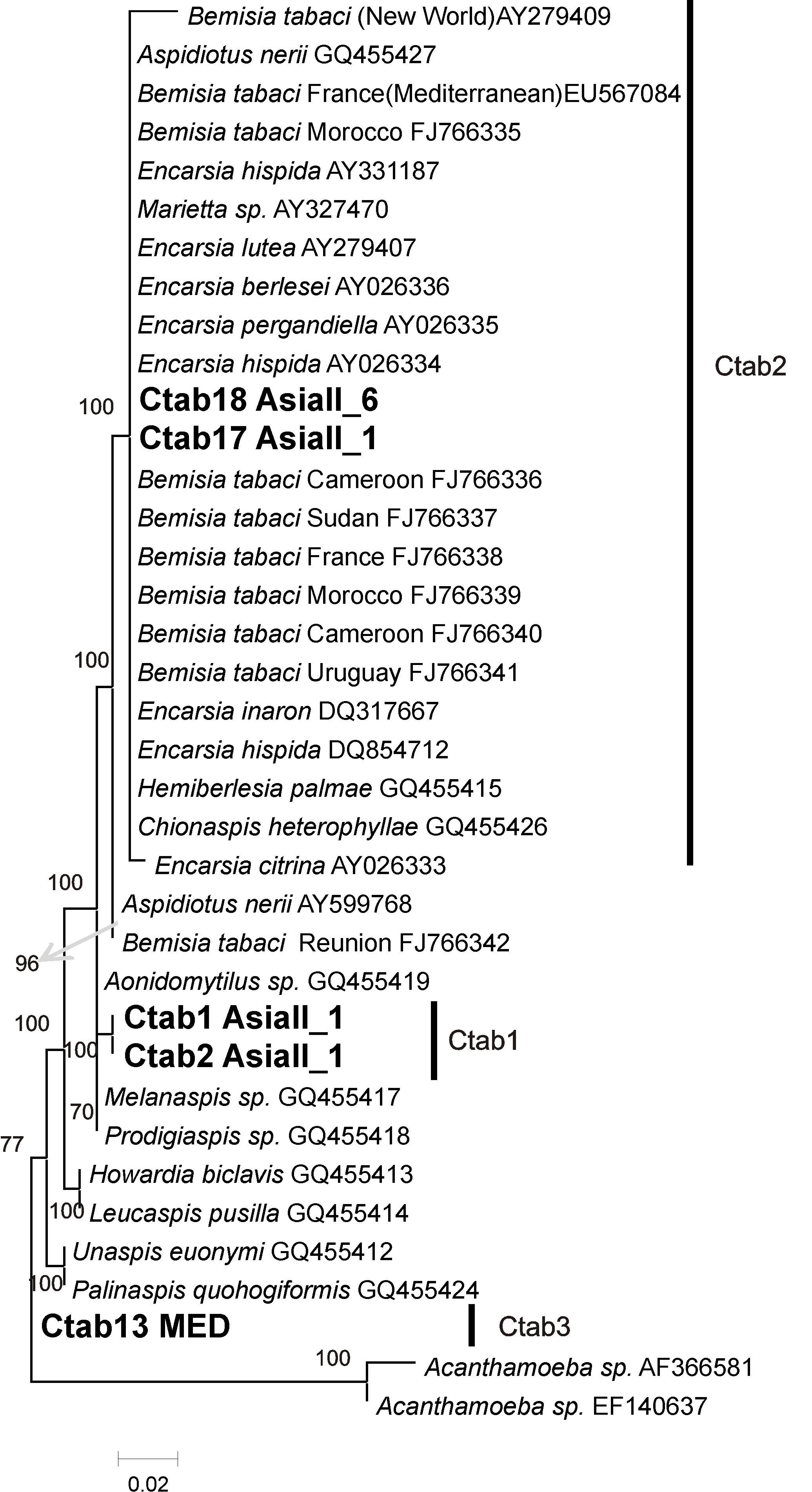
**

**Fig. S1F**

Supplement: Figure S1 — Phylogenetic tree reconstruction of B. tabaci and its endosmbionts based on various gene sequences. A. Phylogenetic tree reconstruction based on mtCOI gene sequences (length = 830 bp) of host B. tabaci cryptic species using maximum-likelihood analysis under the HKY+G substitution model. The bootstrap values are indicated. B. Phylogenetic tree reconstruction based on 16S rRNA gene sequences (length = 1100 bp) of P-endosymbionts of B. tabaci cryptic species using maximum-likelihood analysis under the JC+G substitution model. The bootstrap values are indicated. C. Phylogenetic tree reconstruction based on wsp gene sequences (length = 480 bp) of Wolbachia of B. tabaci cryptic species using maximum-likelihood analysis under the T92+G substitution model. The bootstrap values are indicated. D. Phylogenetic tree reconstruction based on ftsZ gene sequences (length = 850) of Wolbachia of B. tabaci cryptic species using maximum-likelihood analysis under the TN93+G+I substitution model. E. Phylogenetic tree reconstruction based on 23S rRNA gene sequences (length = 550 bp) of Arsenophonus of B. tabaci cryptic species using maximum-likelihood analysis under the HKY+G substitution model. The bootstrap values are indicated. F. Phylogenetic tree reconstruction based on 16S rRNA gene sequences (length = 400) of Cardinium of B. tabaci cryptic species using maximum-likelihood analysis under the K2+G substitution model. The bootstrap values are indicated. (DOC) [file pone.0053084.s001.doc]

**Fig. S2**

**A**


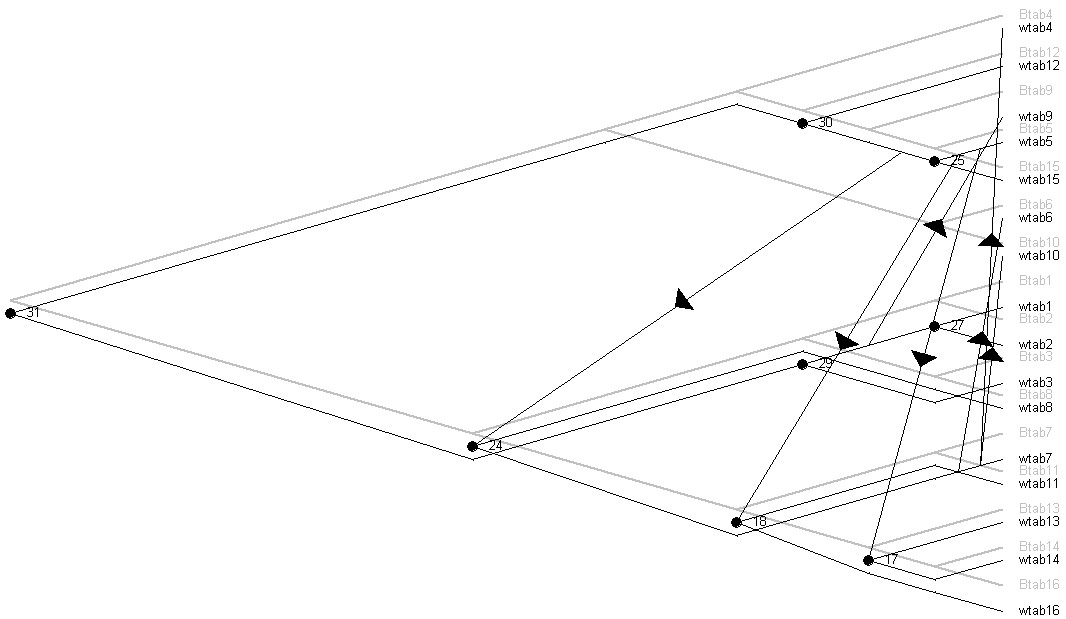


**B**


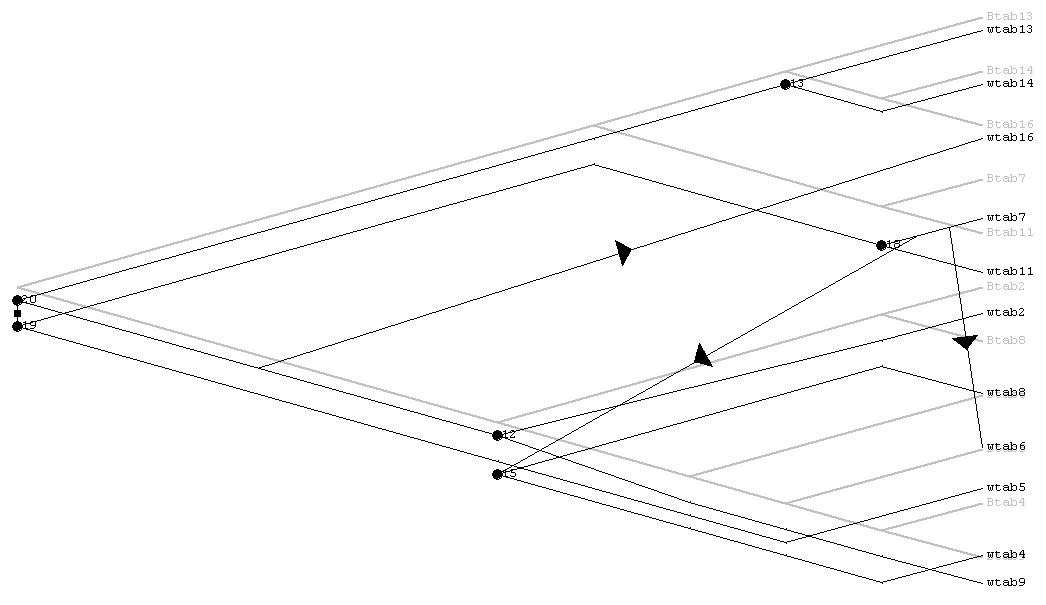


**C**


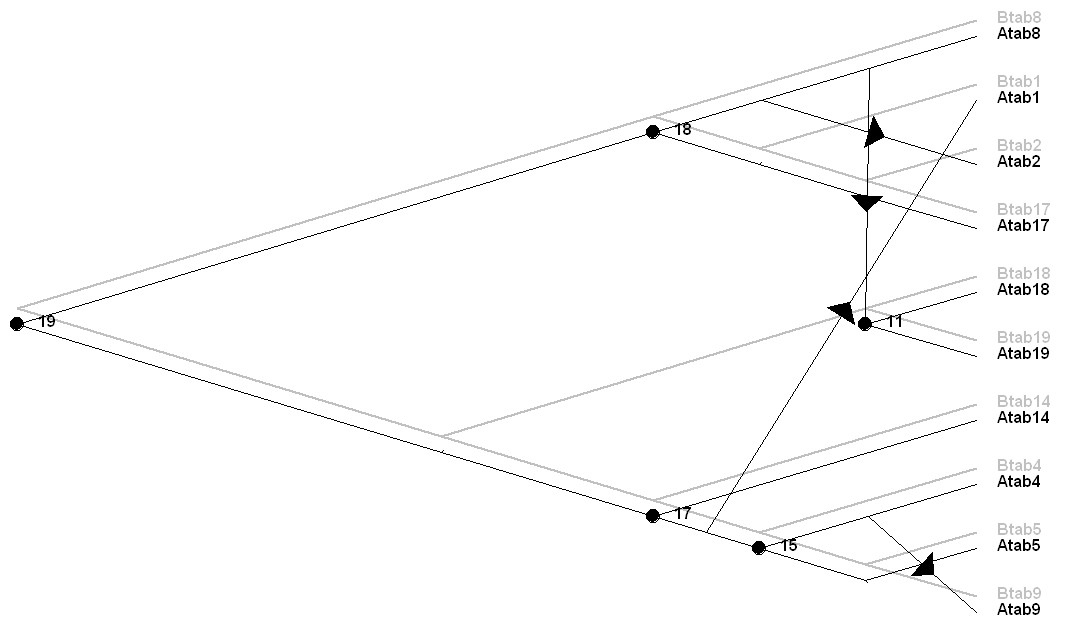


**D**


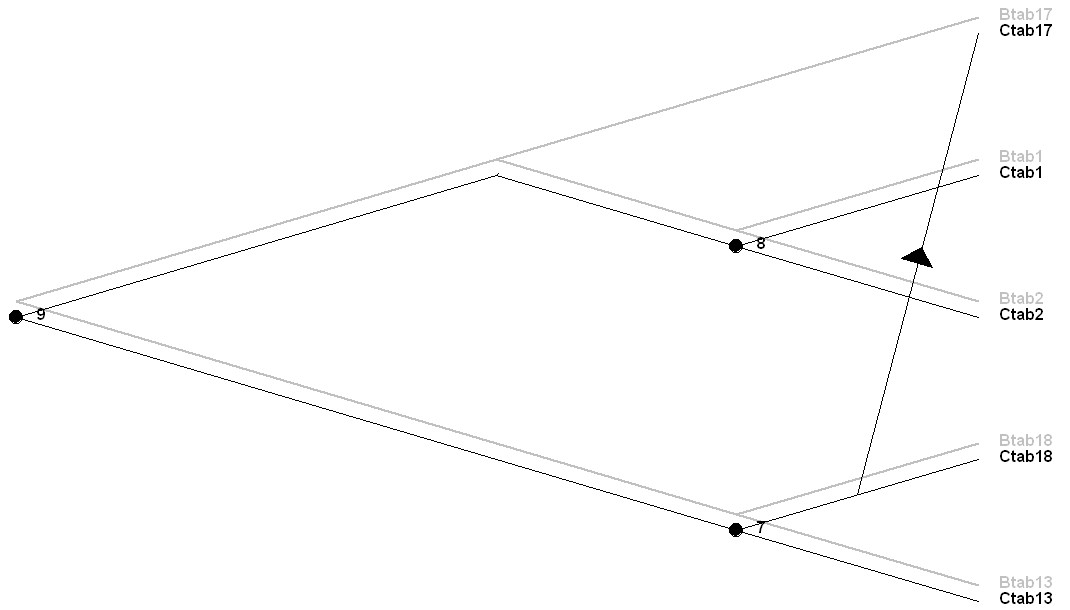


**E**

Supplement: Figure S2 — The exact search at best reconstructions using TreeMap 2.0b show the ML phylogenetic comparison of endosymbionts over their hosts. Endosymbionts displayed in black and the hosts in light grey; in reconstruction; cospeciation events are shown by (•), duplications by(▪) and host switches by (→). A. Host B. tabaci and its P-endosymbionts. B. Host B. tabaci and its Wolbachia (wsp). C. Host B. tabaci and its Wolbachia (ftsZ). D. Host B. tabaci and its Arsenophonus (23S). E. Host B. tabaci and its Cardinium (16S). (DOC) [file pone.0053084.s002.doc]
