# Supplementary material for: Evidence for Horizontal Transmission of Secondary Endosymbionts in the Bemisia tabaci Cryptic Species Complex
Source: PLoS One. 2013 Jan 7;8(1):e53084. doi: 10.1371/journal.pone.0053084 (PMC3538644; doi:10.1371/journal.pone.0053084)
Supplement: Table S1 — Asian B. tabaci cryptic species population sampled for S-endosymbionts. (DOC) [file pone.0053084.s003.doc]

**Table S1** Asian *B. tabaci* cryptic species population sampled for S-endosymbionts.

| **Populations of Cryptic Species** | ***Wolbachia*** | | ***Arsenophonus*** | | ***Cardinium*** | | **Host plants and its family** | | **Geographical Locations** |
| --- | --- | --- | --- | --- | --- | --- | --- | --- | --- |
|  | **n*** | **I**** | **n** | **I**** | **n*** | **I**** | **Scientific name (common name)** | **Family** |  |
| AsiaI | 10 | 9 | 10 | 5 | 10 | 0 | *Ageratina adenophora* (Crofton weed) | Asteraceae | Guangdong, China |
| AsiaI | 10 | 8 | 10 | 4 | 10 | 0 | *Cucurbita Moschata* (Squash) | Cucurbitaceae | Taiwan, China |
| AsiaI | 10 | 7 | 10 | 3 | 10 | 0 | *Glycine max* (Soybean) | Fabaceae | Guangdong, China |
| AsiaI | 10 | 9 | 10 | 5 | 10 | 0 | *Lycopersicon esculentum* (Tomato) | Solanaceae | Yunnan, China |
| AsiaI | 10 | 8 | 10 | 4 | 10 | 0 | *Gossypium herbaceum* (Cotton) | Malvaceae | Guangxi, China |
| AsiaII_1 | 10 | 7 | 10 | 4 | 10 | 2 | *Vicia faba* (Horse Bean) | Fabaceae | Guangdong, China |
| AsiaII_1 | 10 | 6 | 10 | 5 | 10 | 2 | *Vicia faba* (Horse Bean) | Fabaceae | Guangdong, China |
| AsiaII_1 | 10 | 6 | 10 | 5 | 10 | 2 | *Ipomoea batatas* (Sweet potato) | Convolvulaceae | Guangdong, China |
| AsiaII_6 | 10 | 9 | 10 | 3 | 10 | 3 | *Lycopersicon esculentum* (Tomato) | Solanaceae | Yunnan, China |
| AsiaII_6 | 10 | 9 | 10 | 4 | 10 | 3 | *Solanum melongena* (Eggplant) | Solanaceae | Yunnan, China |
| AsiaII_7 | 10 | 8 | 10 | 2 | 10 | 0 | *Codiaeum variegatum* (Garden croton) | Euphorbiaceae | Guangdong, China |
| AsiaII_7 | 10 | 9 | 10 | 3 | 10 | 0 | *Euphorbia pulcherima* (Poinsettia) | Euphorbiaceae | Taiwan, China |
| China1 | 10 | 8 | 10 | 0 | 10 | 0 | *Retinervus Luffae Fructus* (Towel Gourd) | Cucurbitaceae | Hainan, China |
| China1 | 10 | 7 | 10 | 0 | 10 | 0 | *Ipomoea batatas* (Sweet potato) | Convolvulaceae | Guangdong, China |
| MEAM1 | 10 | 5 | 10 | 0 | 10 | 0 | *Glycine max*(Soybean) | Fabaceae | Guangdong, China |
| MEAM1 | 10 | 6 | 10 | 0 | 10 | 0 | *Ageratina adenophora* (Crofton weed) | Asteraceae | Henan, China |
| MED | 10 | 7 | 10 | 6 | 10 | 3 | *Solanum melongena* (Eggplant) | Solanaceae | Yunnan, China |
| MED | 10 | 9 | 10 | 7 | 10 | 3 | *Parthenocissus tricuspidata* (Boston ivy) | Vitaceae | Yunnan, China |
| MED | 10 | 8 | 10 | 5 | 10 | 2 | *Lycopersicon esculentum* (Tomato) | Solanaceae | Yunnan, China |

n*Total number of individuals subjected to PCR amplification, **I Total number of individuals detected as positive for respective infections in its host
